# High prevalence of chronic kidney disease in a community survey of urban Bangladeshis: a cross-sectional study

**DOI:** 10.1186/1744-8603-10-9

**Published:** 2014-02-20

**Authors:** Shuchi Anand, Masuma Akter Khanam, Juliann Saquib, Nazmus Saquib, Tahmeed Ahmed, Dewan S Alam, Mark R Cullen, Michele Barry, Glenn M Chertow

**Affiliations:** 1 Stanford University School of Medicine, 291 Campus Dr, Stanford, CA 94305, USA; 2 International Center for Diarrheal Disease Research, 68 Shaheed Tajuddin Ahmed Sharani, Mohakhali, Dhaka 1212, Bangladesh; 3 Division of Nephrology, Stanford University School of Medicine, 777 Welch Road, Suite DE, Palo Alto, CA 94304, USA; 4 Centre of Clinical Epidemiology and Biostatistics, School of Medicine and Public Health, University of Newcastle, Australia

**Keywords:** Chronic kidney disease, Albuminuria, Insulin resistance, South Asia, Low-income countries

## Abstract

**Background:**

The burden of chronic kidney disease (CKD) will rise in parallel with the growing prevalence of type two diabetes mellitus in South Asia but is understudied. Using a cross-sectional survey of adults living in a middle-income neighborhood of Dhaka, Bangladesh, we tested the hypothesis that the prevalence of CKD in this group would approach that of the U.S. and would be strongly associated with insulin resistance.

**Methods:**

We enrolled 402 eligible adults (>30 years old) after performing a multi-stage random selection procedure. We administered a questionnaire, and collected fasting serum samples and urine samples. We used the Chronic Kidney Disease Epidemiology Collaboration (CKD-EPI) equation to estimate glomerular filtration rate, and sex-specific cut offs for albuminuria: > 1^.^9 mg/mmol (17 mg/g) for men, and >2^.^8 mg/mmol (25 mg/g) for women. We assessed health-related quality of life using the Medical Outcomes Study Short Form-12 (SF-12).

**Results:**

A total of 357 (89%) participants with serum samples comprised the analytic cohort. Mean age of was 49^.^5 (± 12^.^7) years. Chronic kidney disease was evident in 94 (26%). Of the participants with CKD, 58 (62%) had albuminuria only. A participant with insulin resistance had a 3^.^6-fold increase in odds of CKD (95% confidence interval 2^.^1 to 6^.^4). Participants with stage three or more advanced CKD reported a decrement in the Physical Health Composite score of the SF-12, compared with participants without CKD.

**Conclusion:**

We found an alarmingly high prevalence of CKD—particularly CKD associated with insulin resistance—in middle-income, urban Bangladeshis.

## Background

Until recently, most experts presumed that glomerulonephritis or interstitial nephritis were predominant reasons for chronic kidney disease (CKD) in low-and middle-income regions including South Asia [1]. But with an aging population and rapid urbanization, obesity and type two diabetes mellitus (T2DM) afflicts a growing proportion of adults [2,3]. In fact, T2DM already affects nearly 10% of the South Asian population, a burden that approaches that of the U.S., and one that will likely rise at a much faster rate over the next two decades [4]. The prevalence of CKD, particularly CKD related to insulin resistance, will rise in parallel but has been understudied.

Few [5-7] studies have examined the prevalence of CKD in South Asia: most have not used a standard definition of CKD, nor have they assessed albuminuria. In addition, the populations surveyed have not been those at highest risk (i.e., middle income, urban adults who have greater access to energy-dense processed or fast foods). Urban adults have also adopted a sedentary lifestyle, owing in part to the changing nature of their employment, crowding in residential areas, and the ubiquity of televisions [8].

To determine the prevalence of chronic diseases in a high-risk group, we performed a cross-sectional multi-stage random sampled survey of adults living in a middle-income neighborhood in Dhaka, Bangladesh. We hypothesized that the prevalence of CKD would approach that of the U.S., and that a majority of participants with CKD would possess features of insulin resistance.

## Methods

We performed a cross-sectional survey of 402 adult residents of Dhaka to examine the prevalence of obesity and chronic diseases, including T2DM, hypertension, and CKD. This multistage cluster random sampled survey consisted of: 1. a patient questionnaire, 2. anthropometric measurements, and 3. fasting serum sample and urine collection. The Institutional Review Board at Stanford University and the Ethical Review Committee at International Center for Diarrheal Diseases Research, Bangladesh (ICDDR,B) approved the study.

### Survey design

We obtained a detailed map of Mohammedpur, an urban residential area organized in the 1950s within Dhaka with a population of about 450,000 people living in 100,000 households [9]. Of the six blocks that comprise the district, we randomly selected three. We then assigned a study identification number to all of the buildings in the three blocks, and used random sample generator software (SPSS, Chicago, IL) to create a list of 500 buildings. We selected extra buildings to ensure enrollment of one person per building. Most buildings in Mohammedpur are apartment complexes. On arrival at a selected building, the survey team—made up of a research assistant, study nurse and study supervisor—counted the total number of households, and utilized a random number generator to select a single household. If the household members declined to participate or there was non-response, the survey team randomly selected another household within the same building.

Eligible participants were at least 30 years old; if there were more than one eligible participant in the selected household, we randomly selected one from all eligible participants using the same random number generator. We targeted equal enrollment of men and women in the 30 to 50 and > 50 year age category (i.e., 100 individuals in each category). We enrolled participants until the quota for any given combination was reached, after which participants were enrolled only into the remaining strata.

### Data collection

After obtaining informed consent, research assistants administered a patient questionnaire in Bangla. Using a structured questionnaire, we obtained data on: socioeconomic status including education and occupation, tobacco use, self-reported personal and family history of chronic diseases, diet and physical activity, and family planning. We administered the Medical Outcomes Study Short-Form-12 version two questionnaire (SF-12) to assess health related quality of life (HRQoL). At the completion of the patient questionnaire, the research assistant made an appointment for a second visit (within one week) to obtain a urine specimen, fasting serum sample, and anthropometric measurements.

A study nurse measured height, weight, and waist and hip circumference on all participants using standardized procedures [10]. The nurse also obtained two blood pressure readings using a manual sphygmomanometer after the participant had remained in a seated position for five minutes with his or her arm resting on the chair. The nurse used standard phlebotomy procedures to obtain a fasting serum sample and a urine specimen.

### Laboratory analyses

We transported serum and urine samples to the ICDDR,B central laboratory within one hour of collection. We processed all samples for serum creatinine, hemoglobin A1c, and fasting glucose. Due to budgetary limitations, we performed more expensive tests—urine albumin and creatinine, and serum lipids and triglycerides—in a select group of participants. This group (n = 341) fit at least one criteria for metabolic syndrome, had urine dipstick positive for proteinuria, or had risk factors for CKD (i.e., self-reported personal or family history of diabetes, hypertension, or CKD, or self-reported personal history of auto-immune disease, kidney stones, or birth weight below 2^.^5 kg) [11].

Urine albumin was measured by immunoturbidimetric assay using Olympus AU 640, and plasma and urinary creatinine were measured by enzymatic colorimetric assay using Olympus AU 640 (Olympus corporation, Tokyo, Japan). Assay variation was checked daily with an internal quality assessment scheme, and the coefficient of variation for each test was below 5%.

### Definitions of chronic diseases and risk factors

We defined a participant as having T2DM if he or she had fasting blood glucose ≥ 7^.^0 mmol/L or hemoglobin A1c ≥ 6^.^5% or medication use for self-reported diabetes. We defined metabolic syndrome according to International Diabetes Federation criteria [12] as centripetal obesity (waist circumference ≥ 90 cm for men and ≥ 80 cm for women) plus any two of the followings: (1) triglycerides > 1^.^7 mmol/L or treatment for elevated triglycerides, (2) HDL cholesterol < 1^.^03 mmol/L in men or < 1^.^29 mmol/L in women, or treatment for low HDL, (3) systolic blood pressure > 130 mmHg, diastolic blood pressure > 85 mmHg, or treatment for hypertension, and (4) fasting plasma glucose > 5^.^6 mmol/L. We defined a participant as having insulin resistance if he or she met criteria for T2DM or metabolic syndrome. We used the following categories for Quételet’s (body mass) index (BMI): < 25 (lean or normal), 25 to < 30 (overweight), and ≥ 30 kg/m^2^ (obese). We captured leisure time physical activity by the following question: 'How often do you walk outside the home for more than 10 minutes without stopping?’ We categorized the responses as 'Rarely,’ 'Weekly’, or 'Daily.’

We defined a participant as having cardiovascular disease if he or she reported using medications for blood pressure or heart disease, or had systolic blood pressure ≥ 140 mmHg and diastolic blood pressure ≥ 90 mmHg.

### Definition of CKD

We used the Chronic Kidney Disease Epidemiology Collaboration (CKD-EPI) equation to estimate glomerular filtration rate (eGFR), and the National Kidney Foundation Kidney Disease Outcomes Quality Initiative (NKF/KDOQI) guidelines to define and stage CKD [11]. We used a sex- specific cut off for albuminuria: ≥ 1^.^9 mg/mmol (17 mg/g) creatinine for men, and ≥ 2^.^8 mg/mmol (25 mg/g) creatinine for women. Thus we defined a participant as having CKD if he or she had eGFR below 60 ml/min/1.73 m^2^ or had albuminuria. We also present prevalence results for albuminuria and CKD using the ≥ 3^.^4 mg/mmol (30 mg/g) creatinine cutoff.

### Statistical analyses

We present continuous data as mean (± standard deviation) and categorical data as proportions, with comparisons *via* Student’s t-test or Pearson’s χ^2^ test, as appropriate. We stratified by sex and performed logistic regression to test the associations among potential covariates including education, wealth status, physical activity, tobacco use, measures of body size (BMI, waist circumference, and waist-to-hip ratio), and presence of insulin resistance or cardiovascular disease. We used generalized linear models to evaluate the association between CKD and the Physical and Mental Health Composite t-scores on the SF-12. We used the New England Medical Center (NEMC) guidelines to score the SF-12 [13]. The primary analysis evaluated these associations using NKF/KDOQI stages of CKD, calculated based on eGFR and/or albuminuria. In multivariable analyses, we adjusted for the residual effects of age and sex, as well as for insulin resistance. Since age and sex are embedded in the eGFR calculation, we performed companion analyses in which we used the reciprocal of serum creatinine as a marker for kidney function. We considered inference tests as significant if the two-tailed p-value was <0^.^05. We conducted all analyses using SAS, v9.3 (SAS Institute Inc., Cary, NC).

## Results

A total of 357 (89%) participants provided serum samples and comprise the analytic cohort; 182 (51%) were men and 175 (49%) were women. Spot urine albumin and creatinine were assessed among 341 (96%) participants. As reported previously, men were more likely to be married, employed, and educated at the high school level (Table 1) compared with women. Men also had lower mean BMI, and were more likely to use cigarettes, whereas women more often consumed smokeless tobacco. Two hundred and twelve (59%) participants had features of insulin resistance, 138 (65%) of whom had T2DM.

**Table 1:** Characteristics of participants with available serum creatinine determinations (n = 357)

	**Men mean ± SD or N (%)**	**Women mean ± SD or N (%)**
Demographics		
Age (in years)	49^.^4 ± 12^.^1	49^.^6 ± 13^.^4
Married*	170 (93)	125 (71)
High school education or more*	96 (53)	53 (30)
Employed*	139 (76)	125 (71)
Wealth index > 3†	33 (18)	34 (19)
Residence in Dhaka (in years)*	30^.^0 ± 16^.^2	33^.^8 ± 15^.^1
Clinical characteristics		
BMI (kg/m^2^) categories		
< 25	92	40
25 to < 30	82	71
≥ 30	51	21
Smoker (current or past)*	107 (59)	5 (3)
Smokeless tobacco user*	30 (17)	45 (26)
Self-reported hypertension*	57 (31)	76 (43)
Measured systolic blood pressure	118^.^4 ± 15^.^7	117^.^8 ± 16^.^6
Measured diastolic blood pressure	74^.^6 ± 11^.^2	74^.^5 ± 13^.^3

*represents significant difference at the p < 0^.^05 level. †A wealth index above 3 indicates owning 3 or more of: apartment/house, car, power source, or air conditioner. *Abbreviations*: BMI-Quételet’s (body mass) index, SD-standard deviation.

### CKD prevalence

**Figure 1:**
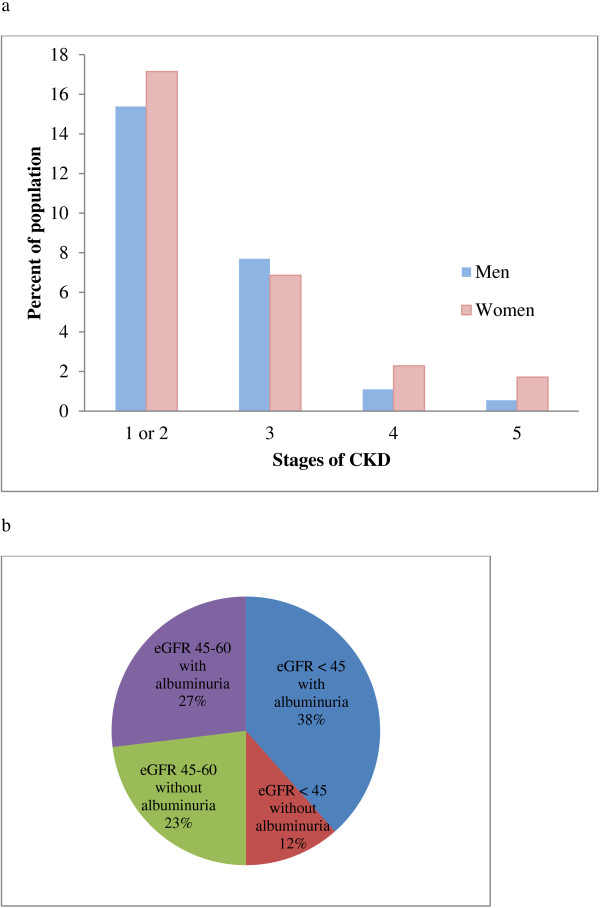
**Stages of CKD with details on albuminuria for stage three CKD. a**. Stages of CKD. Overall 94 (27%) of participants had CKD. Stage one or two CKD (albuminuria with eGFR > 90 or between 60–90 ml/min/1.73 m^2^) was present in 58 (62%). Stage five CKD was present in 4 (4%). Abbreviations: CKD-Chronic kidney disease, eGFR-estimated glomerular filtration rate. **b**. Characterizing stage three CKD. Among participants with stage three CKD (n = 26, 28%), 6 (23%) had an isolated reduction in eGFR between 45–59 ml/min/1.73 m^2^; all others had either stage 3b CKD or accompanying albuminuria. Abbreviations: CKD-chronic kidney disease; eGFR-estimated glomerular filtration rate.

**Figure 2:**
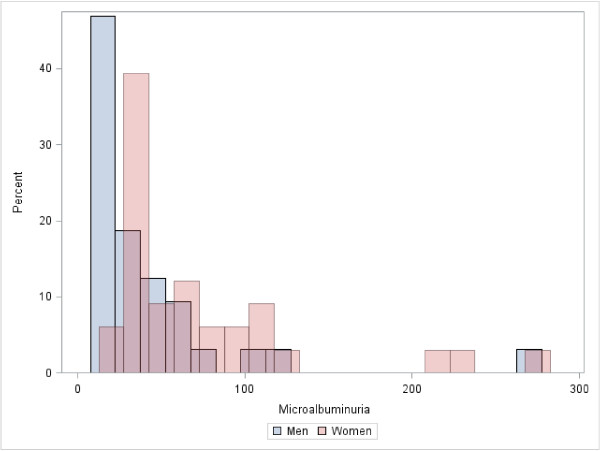
**Distribution of microalbuminuria in men and women.** Microalbuminuria was defined as 1.9-34 mg/mmol (17 – 300 mg/g) creatinine for men and (2.8-34 mg/mmol (25 – 300 mg/g) creatinine for women. There were a total of 79 (22%) cases of albuminuria, 65 (83%) of which were in the microalbuminuria range.

Ninety-four (26%) participants met the definition of CKD (Figure 1a and b), 45 men and 49 women. A majority (n = 58 (62%)) had stage one or two CKD (i.e., albuminuria only). Among the 36 participants with stage three or more advanced CKD, 21 (58%) had associated albuminuria. Overall, there were 79 (22%) participants with albuminuria, 65 (83%) of whom had microalbuminuria (Figure 2). Using an albuminuria cutoff of ≥ 3.4 mg/mmol creatinine, 56 and 72 (20%) participants were classified as having albuminuria and CKD respectively.

### Clinical correlates of CKD in Bangladesh

On unadjusted analyses, education level was not associated with the presence of CKD among men, although among women, those who had received fewer than 5 years of education experienced higher odds of CKD compared with women educated at the University level or beyond (Odds ratio [OR] 3^.^6, 95% Confidence Interval [CI] 1^.^4 to 9^.^4). Wealth status was not associated with CKD among women or men. Since smokers in our sample were predominantly (96%) men, we tested the association between smoking and CKD among men only and found no association. Use of smokeless (chewable) tobacco was associated with higher odds of CKD among women (OR 2^.^8, 95% CI 1^.^4 to 5^.^7).

Larger waist circumference and waist-to-hip ratio (using International Diabetes Foundation criteria) [12], BMI, and leisure-time physical activity were not significantly associated with CKD.

### CKD and insulin resistance

**Figure 3:**
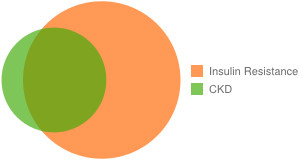
**Overlap between insulin resistance and chronic kidney disease.** Of the 94 participants with CKD, 75 (79%) were among participants with insulin resistance, defined as metabolic syndrome or T2DM. Of these, 59 (63%) had T2DM. Abbreviations: CKD—chronic kidney disease, T2DM-type two diabetes mellitus.

A majority of participants with CKD also had features of insulin resistance (Figure 3). The unadjusted odds of CKD among participants with insulin resistance were 3^.^6 fold higher (95% CI 2^.^1 to 6^.^4) than among participants without insulin resistance. Upon stratification by sex, the odds of CKD among men with insulin resistance were 3^.^1 (95% CI 1^.^5 to 6^.^4) and among women were 4^.^7 (95% CI 1^.^9 to 11^.^9). Cardiovascular disease was also evident in more than two-thirds (n = 64, 68%) of participants with CKD. The measured average blood pressure of a participant with CKD was 125/78 mmHg, approximately 10/5 points higher than in participants without CKD (p <0^.^001 and 0^.^005, for systolic and diastolic pressure, respectively).

### CKD and HRQoL

Mean t-scores on the SF-12 NEMC Physical Health Composite t-Score (PCS) were lower among participants with CKD (Table 2). There was no significant difference in mean t-scores on the SF-12 Mental Health Composite Score (MCS), although the mean MCS for participants with stage five CKD was significantly lower than for all other stages. Multivariable analyses adjusting for the residual effects of age and gender as well as for insulin resistance showed that mean scores on PCS were significantly lower among participants with stage three or more advanced CKD.

**Table 2:** SF-12 scores among participants with chronic kidney disease

	**PCS Mean ± SE**	**MCS Mean ± SE**
*Unadjusted model*		
No chronic kidney disease (ref)	40.2 ± 0.6	41.1 ± 0.7
Chronic kidney disease (n = 94)		
Stage 1 or 2 (n = 58)	**38.6 ± 1.3**	43.0 ± 1.2
Stage 3 (n = 26)	**34.9 ± 2.0**	45.6 ± 1.7
Stage 4 (n = 6)	**29.2 ± 2.0**	32.9 ± 6.2
Stage 5 (n = 4)	**27.6 ± 2.2**	**23.3 ± 4.2**
Adjusted model*		
No chronic kidney disease (ref)		
Chronic kidney disease	39.8 ± 0.5	40.9 ± 0.7
Stage 1 or 2		
Stage 3	39.1 ± 1.1	43.3 ± 1.4
Stage 4	**36.6 ± 1.8**	Up
Stage 5	**32.2 ± 3.5**	34.6 ± 4.6

Values in bold are significantly different from the referent category. *Adjusted for insulin resistance, and residual effects of age and gender. Stages are based on the National Kidney Foundation/Kidney Disease Outcomes Quality Initiative definition of chronic kidney disease. *Abbreviations*: PCS: New England Medical Center Physical Health Score, MCS: Mental Health Score, SE: standard error.

On companion analyses using the reciprocal of serum creatinine as a main exposure of interest (as opposed to eGFR), we found that the PCS was inversely correlated with serum creatinine and age, and was lower among women compared with men.

## Discussion

Among randomly sampled adults living in a middle-income neighborhood of Bangladesh, approximately one in five were found to have CKD. Nearly four of five participants with CKD had features of insulin resistance, which was the predominant risk factor linked with CKD in our study. In contrast to data from the U.S. and other countries where stage three CKD predominates [14-16], we found that stage one or two CKD (albuminuria only) was most common. The high prevalence of insulin resistance and associated albuminuria among our participants with CKD implies that they are at high risk for progression of CKD and for experiencing a cardiovascular event.

No prior data exist on prevalence of CKD in Bangladesh. Two studies from New Delhi, India have used variable definitions of CKD. A study of 4,712 adults living in South Delhi found that 0.8% had serum creatinine above 159 umol/L (1.8 mg/dL) [5]; the comparable percentage in our study was 3%. Another study of 5,252 adults also based in Delhi reported a prevalence of reduced eGFR (below 60 ml/min/1.73 m^2^) equal to 4% [6]; the comparable percentage in our study was 10%. The Thai-Global Screening and Early Evaluation of Kidney Disease (SEEK) study sampled 3,459 from 10 provinces. Both albuminuria and serum creatinine were evaluated and the standard NKF/KDOQI definition was applied; 17% of surveyed participants met criteria for CKD [17]. As reference, the most recent estimates of CKD prevalence from Norway [18], Spain [15], U.S. [14], and Japan [16] are between 10 and 13% of the adult population.

We hypothesize three reasons for the higher prevalence of CKD noted in our study. First, we surveyed adults over 30 years old, whereas Thai-SEEK and NHANES enrolled adults over 18 and 20 years old, respectively. Second, by surveying a well-established neighborhood of a major urban hub, we are sampling from a population at higher risk, not representative of the risk experienced by people living in rural areas or poorer persons. Migrating to an urban area was associated with an approximately 1^.^5-fold increase in odds of a sedentary lifestyle, three-fold increase in odds of obesity, and two-fold increase in odds of T2DM among men working in factories in India, compared with their rural counterparts [3]. In low-income countries, the prevalence of obesity increases with increasing wealth, although this trend may soon arrest or reverse as prices of fruits and vegetables climb while processed foods become cheaper and more easily accessible [19].

A majority of participants who were identified as having CKD had stage one or two CKD (i.e., albuminuria only). As we did not repeat assessment of albuminuria, we do not have population-specific data on persistent albuminuria. However extrapolating data from NHANES on spot *versus* persistent albuminuria, 51% of participants with microalbuminuria and stage one CKD and 75% of participants with microalbuminuria and stage two CKD would be estimated to have *persistent* microalbuminuria, yielding an adjusted (approximate) CKD prevalence equal to 19%, rather than 26%. Thus, the prevalence of CKD in the urban Bangladeshi population would still be higher than that in the U.S., Europe, and Japan. Strikingly, we identified four persons (1%) with stage five CKD (eGFR below 15 ml/min/1.73 m^2^). If we extrapolate only to the >30 year old population of Mohammedpur, a stage five CKD prevalence of 1% would imply that roughly 1,800 persons would be at risk for imminent kidney failure in a district that represents 0.3% of the Bangladeshi population. Even these 1,800 persons represent an immense burden in a country where kidney transplants are performed in only one and dialysis in just four of 13 government hospitals [20].

Aside from the need for transplantation or dialysis, persons with CKD (especially those with albuminuria) experience an increased risk for cardiovascular disease and mortality. Microalbuminuria, found in 70% of our participants with CKD, has been linked to a 30 to 80% increase in the risk for all-cause and cardiovascular mortality; overt proteinuria, found in 15% of our participants with CKD, can double or triple these risks [21]. The majority of our participants with CKD also had T2DM, which increases risk for all-cause and cardiovascular mortality 20 to 90%, relative to persons with CKD who do not have T2DM [1].

In Bangladesh, care for chronic illnesses is not yet in line with the care for infectious diseases; much of the public sector health budget is devoted to treating episodic illnesses such as diarrhea or respiratory illness. No national guidelines exist for primary health care screening for chronic illnesses such as CKD. Even among patients identified to have a chronic illness, cost of treatment is a major burden: while the government subsidizes some of the care (e.g., the doctor’s visit) in the public sector, patients may still pay for other aspects (e.g., medications). Some subsidies are available from social welfare organizations. Those who can afford it do seek private care, and a negligible percent goes abroad for better treatment.

We also found that moderate to advanced CKD was associated with a decrement in self-reported physical health. While few studies have explored HRQoL in patients with non-dialysis requiring CKD, the associated decrement in self-reported physical health seen among participants with moderate to advanced CKD in this study is considered to be clinically meaningful [22]. Thus, if the burden of CKD is not lessened in the coming decades, we can anticipate higher rates of cardiovascular disease, frailty, and debility in the urban Bangladeshi population, even in late middle and early older age.

Our study has several strengths. We adopted a random sampling procedure in this community-based survey. Trained and experienced nurses and research assistants used standardized protocols for physical measurements. Anticipating low rates of awareness of chronic diseases, we employed laboratory measurement to diagnose T2DM, metabolic syndrome, and CKD. Ours is one of few studies in the region to include an assessment of albuminuria and HRQoL.

However, given our modest sample size and that our survey was limited to a particular urban neighborhood, we cannot extrapolate our findings to all of Bangladesh. Our modest sample size also limited our assessment of the correlates of CKD. Ideally, repeat measures of serum creatinine and urinary albumin excretion might allow us to be more precise in our prevalence estimates.

## Conclusions

Our study points to an alarmingly high rate of CKD—roughly one in five—among urban middle-income Bangladeshis. The kidney disease we detected will pose a significant clinical burden to this resource-poor setting with limited healthcare infrastructure and very limited capacity to manage advanced and end-stage disease. Additional studies are required to define CKD prevalence, incidence rates, and region- and population-specific risk factors in low- and middle-income countries around the globe, as well as an urgent need to design low-cost programs to detect and manage CKD in Bangladesh and other countries in South Asia.

## Abbreviations

CKD: Chronic kidney disease; CKD-EPI: Chronic kidney disease epidemiology collaboration; CI: Confidence interval; eGFR: Estimated glomerular filtration rate; HRQoL: Health-related quality of life; ICDDR,B: International center for diarrheal disease research, Bangladesh; SF-12: Medical outcomes study short form-12; MCS: Mental health composite t-score; NKF/KDOQI: National kidney foundation/kidney disease outcomes quality initiative; NHANES: National health and nutrition examination survey; NEMC: New England medical center; OR: Odds ratio; PCS: Physical health composite t-score; BMI: Quételet’s (body mass) index; SEEK: Screening and early evaluation of kidney disease; T2DM: Type 2 diabetes mellitus.

## Competing interests

The authors have no conflicts of interest to declare. This work has not been published elsewhere.

## Authors’ contributions

Dr. SA was helped with survey design, and data collection and analysis, and performed the manuscript write-up. Dr. AK led survey design, and data collection. Drs JS, TA, and DSA helped with survey design, and data collection and analysis, and provided editorial input for manuscript write-up. Drs MB, MRC, and GMC helped secure funding for data collection, advised survey design, and provided editorial input for manuscript write-up. All authors read and approved the final manuscript.
